# Post-Inhaled Corticosteroid Pulmonary Tuberculosis Increases Lung Cancer in Patients with Asthma

**DOI:** 10.1371/journal.pone.0159683

**Published:** 2016-07-22

**Authors:** Zhi-Hong Jian, Jing-Yang Huang, Frank Cheau-Feng Lin, Oswald Ndi Nfor, Kai-Ming Jhang, Wen-Yuan Ku, Chien-Chang Ho, Chia-Chi Lung, Hui-Hsien Pan, Min-Chen Wu, Ming-Fang Wu, Yung-Po Liaw

**Affiliations:** 1 Department of Public Health and Institute of Public Health, Chung Shan Medical University, Taichung City, Taiwan; 2 School of Medicine, Chung Shan Medical University, Taichung City, Taiwan; 3 Department of Thoracic Surgery, Chung Shan Medical University Hospital, Taichung City, Taiwan; 4 Department of Neurology, Changhua Christian Hospital, Changhua, Taiwan; 5 Department of Physical Education, Fu Jen Catholic University, New Taipei City, Taiwan; 6 Department of Family and Community Medicine, Chung Shan Medical University Hospital, Taichung City, Taiwan; 7 Department of Pediatrics, Chung Shan Medical University Hospital, Taichung City, Taiwan; 8 Office of Physical Education, Chung Yuan Christian University, Taoyuan City, Taiwan; 9 Divisions of Medical Oncology and Pulmonary Medicine, Chung Shan Medical University Hospital, Taichung City, Taiwan; University of Athens, GREECE

## Abstract

**Purpose:**

To evaluate the association between post-inhaled corticosteroid (ICS) pulmonary tuberculosis (TB), pneumonia and lung cancer in patients with asthma.

**Methods:**

The study samples were collected from the National Health Insurance Database. Asthmatic patients who were first-time users of ICS between 2003 and 2005 were identified as cases. For each case, 4 control individuals were randomly matched for sex, age and date of ICS use. Cases and matched controls were followed up until the end of 2010. Cox proportional hazard regression was used to determine the hazard ratio for pulmonary infections and lung cancer risk in the ICS users and non-users.

**Results:**

A total of 10,904 first-time users of ICS were matched with 43,616 controls. The hazard ratios for lung cancer were: 2.52 (95% confidence interval [CI], 1.22–5.22; p = 0.012) for individuals with post-ICS TB, 1.28 (95%CI, 0.73–2.26; p = 0.389) for post-ICS pneumonia, 2.31(95%CI, 0.84–6.38; p = 0.105) for post-ICS pneumonia+TB, 1.08 (95%CI, 0.57–2.03; p = 0.815) for TB, 0.99 (95%CI, 0.63–1.55; p = 0.970) for pneumonia, and 0.32 (95%CI, 0.05–2.32; p = 0.261) for pneumonia+ TB, respectively.

**Conclusions:**

Post-ICS TB increased lung cancer risk in patients with asthma. Because of the high mortality associated with lung cancer, screening tests are recommended for patients with post-ICS TB.

## Introduction

Lung cancer is the leading cause of cancer mortality [1]. Although smoking is the major risk factor [2], other factors may also be linked to the increased risk of lung cancer. Recent studies have suggested a strong association between inflammation, infection and lung cancer [3, 4]. Asthma, pulmonary tuberculosis (TB) and pneumonia cause airway inflammation and are also associated with lung cancer [5–7]. The prevalence of asthma (11.9%) in Taiwan is high [8]. Inhaled (ICS) and oral corticosteroids (OCS) are the preferred treatments for airway and systemic inflammation in patients with asthma [9–11].

Minimizing inflammation in the lungs may decrease lung cancer risk. A nested case-control study reported a decreased risk of lung cancer in first-time users of ICS [12]. Moreover, ICS has been associated with a reduced risk of lung cancer in patients with chronic obstructive pulmonary disease (COPD) [13] and COPD who quit smoking [14].

ICS use may suppress the immune system, thereby increasing the risk of TB [15] and pneumonia [16]. TB [17] and pneumonia [7] are risk factors of lung cancer. Co-existing asthma and TB has increased the incidence and mortality of lung cancer [5, 18, 19]. The relationship between pneumonia and TB and lung cancer in asthmatics with ICS use remains unclear. In this study, we evaluated the association between post-ICS pulmonary infections and lung cancer using samples collected from the National Health Insurance Research Database (NHIRD).

## Methods

### Ethics Statement

This retrospective cohort study was conducted using the NHIRD, Taiwan Cancer Registry Database (TCRD), and National Death Registry Database (NDRD). Individual informed consent was waived because the source data was encrypted and the data extracted was anonymous. This study was approved by the Institutional Review Board of the Chung-Shan Medical University Hospital, Taiwan.

### Data Source

The NHIRD included a comprehensive health care information, such as medical diagnoses, prescriptions and information on out and in-patient care. TCRD included demographic characteristics and clinical data, such as age, gender, tumor stage, and primary tumor location. These databases were used to determine the age at diagnosis, person-year follow-up, vital status and potentially unconfirmed cancer diagnoses.

### Asthmatic patients and ICS use

We first identified 1,124,939 patients with asthma from 2001–2005 who were free of lung cancer before 2002 using the NHIRD inpatient and ambulatory care orders. Excluded were patients who received ICS before 2002 (n = 16,601) and those with incomplete information regarding sex and registered data (n = 48,209). We enrolled 19,335 asthmatic patients who received ICS for more than 3 months. Additionally, patients <20 years or >100 years of age were also excluded (n = 1,894).

The ICS included all forms of orally inhaled beclomethasone, budesonide, fluticasone, and ciclesonide whether dispensed alone or in a combination inhaler with an inhaled β2 agonist. Information about the prescription (the dates the drug is prescribed, the number of days of treatment, dosage and the number of refills) were additionally collected. The date of the first use of ICS was referred to as the index date.

### Post- ICS pulmonary TB and pneumonia

A diagnosis of pulmonary TB (International Classification of Diseases, Ninth Revision, Clinical Modification [ICD-9-CM] code: 010–012, 018, and137) was confirmed by either 2 outpatient visits or one hospitalization after the index date. Pneumonia (ICD-9-CM codes: 480–486, and 487.0) was confirmed by one hospitalization. Excluded were individuals who were diagnosed with TB or pneumonia before or within 3 months after the index date (n = 4,690).

### Identification of lung cancer

Lung cancer was identified using the ICD-9-CM code 162. The histologic types of lung cancer were further confirmed using the TCRD. Patients who died and those that had lung cancer within 2 years after the index date were excluded (n = 1,538).

### Study cohorts

**Fig 1:**
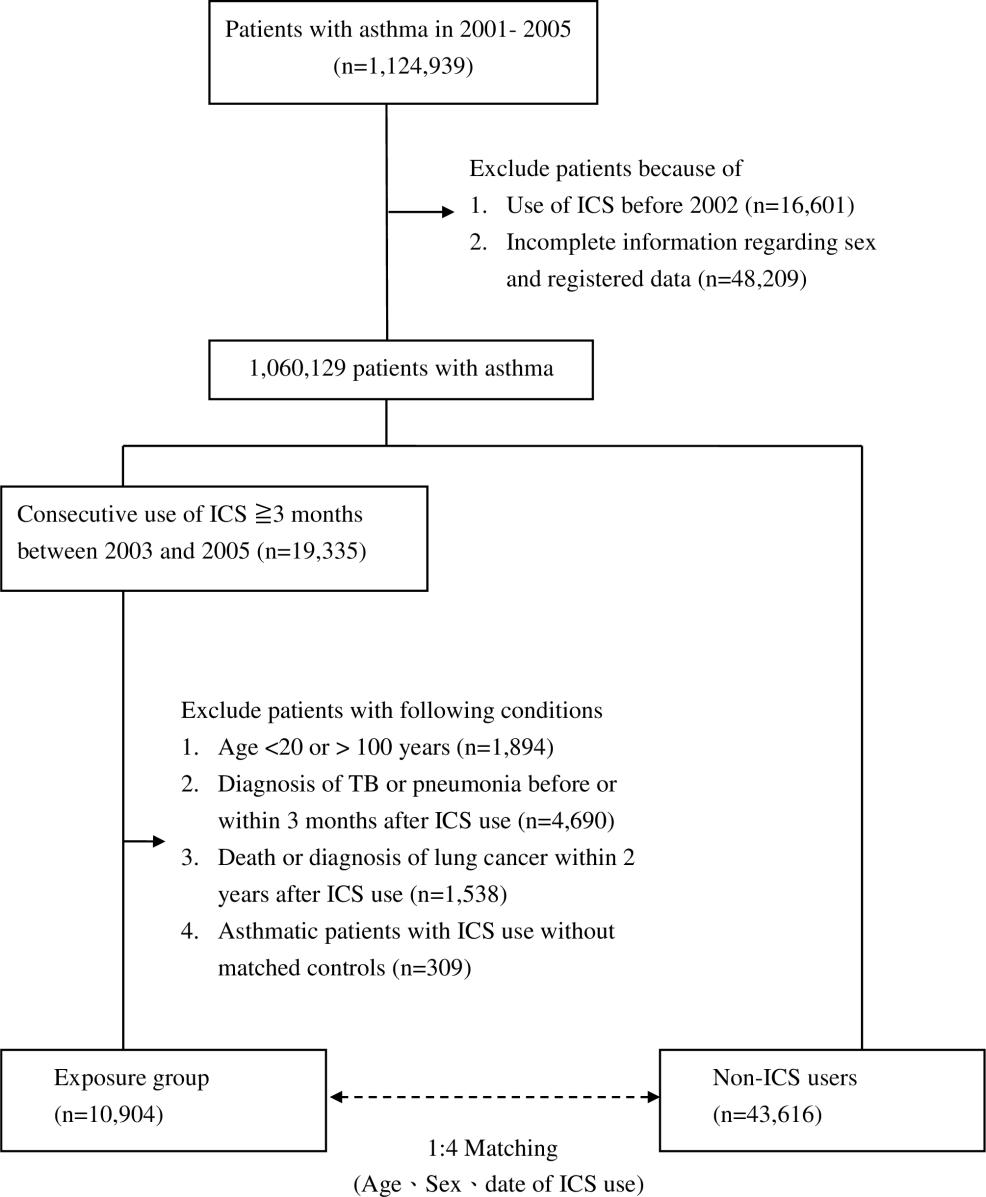
Flow diagram of the enrollment process. ICS, inhaled corticosteroid; TB, tuberculosis.

Asthmatic patients who were first-time users of ICS between 2003 and 2005 were identified as cases. For each case, 4 controls were randomly matched by sex, age and index date from asthmatic patients who did not use ICS. Cases without matched controls were excluded (n = 309). The final analysis included 10,904 cases and 43,616 matched controls (Fig 1). The eligible participants were followed up until the diagnosis of lung cancer, death, loss to follow-up, or the study end in 2010.

### Medications

An OCS user was defined as an individual who took a cumulative dose of 1,680 mg (or 60 mg daily for 4 weeks) of hydrocortisone equivalents or more during the 1-year period after the index date [15]. The OCS prescriptions were converted to hydrocortisone using the following equivalent dose: 4 mg of hydrocortisone = 1 mg of prednisolone = 5 mg of cortisone = 0.8 mg methylprednisolone = 0.8 mg of triamcinolone = 0.4 mg of paramethasone = 0.15mg of betamethasone = 0.15mg of dexamethasone) [20].

The inhaled respiratory medications included short-acting inhaled β2 agonists (SABAs; salbutamol, fenoterol, procaterol, or terbutaline) and long-acting inhaled β2 agonists (LABAs; salmeterol, formoterol, indacaterol, or olodaterol). Other respiratory medications included leukotriene receptor antagonist (LTRA; montelukast) and theophylline.

### Variables of exposure

The pre-existing comorbidities were confirmed by either 2 outpatient visits or one hospitalization in one year. They included asthma (ICD-9-CM: 493), COPD (ICD-9-CM: 490, 491, 492, 494, and 496), chronic kidney disease (ICD-9-CM: 585 and 586), diabetes mellitus (ICD-9-CM: 250), hyperlipidemia (ICD-9-CM: 272), liver cirrhosis (ICD-9-CM: 571.2, 571.5, and 571.6), smoking-related cancers (ICD-9-CM: 140–150, 157, 160–161, and 189), autoimmune disease (ICD-9-CM: 710 and 714), atopic dermatitis (ICD-9-CM: 691), and rhinosinusitis (ICD-9-CM codes: 472.0, 473, and 477). For evaluating asthma severity, we assessed the number of outpatient and inpatients visits for lung diseases within 2 years after the index date. However, personal information including ethnicity, family history, lifestyle, occupation, and habits such as smoking and alcohol use was not available in the database, hence preventing direct adjustment for possible confounders.

### Statistical analysis

The SAS 9.3 software (SAS Institute, Cary, NC) was used for data analysis. Descriptive statistical analyses were conducted using the t-test while chi-square test was used to compare the baseline sociodemographic characteristics and comorbidities between ICS users and their controls. At the univariate level, Kaplan-Meier survival plots and log-rank test were used to evaluate the impact of the predictor variables on lung cancer while Cox proportional hazards regression analysis was used at the multivariate levels. A p-value <0.05 (2-tailed) were considered statistically significant.

## Results

Demographic characteristics, medications, comorbidities, and follow-up durations are shown in Table 1. Patients who used ICS had higher rates of lung cancer, TB, pneumonia, respiratory medication use, COPD, hyperlipidemia, rhinosinusitis, and outpatient and inpatient visits for lung disease than their control counterparts.

**Table 1:** Characteristics of the Study Population.

	ICS		No ICS	P-value
	(N = 10,904)		(N = 43,616)	
**Pulmonary disease combinations (%)**				<0.001
None	10,022 (91.9)		41,227 (94.5)	
TB	152 (1.4)		496 (1.1)	
Pneumonia	642 (5.9)		1,700 (3.9)	
Pneumonia+ TB	88 (0.8)		193 (0.5)	
**No. of lung cancer**	148		469	0.013
**Histologic type of lung cancer (%)**				0.542
Squamous cell carcinoma	32 (21.6)		117 (25.0)	
Adenocarcinoma	63 (42.6)		185 (39.5)	
Small cell carcinoma	18 (12.2)		43 (9.1)	
Others	35 (23.6)		124 (26.4)	
**Follow-up time (person-months)**	5.5×10^5^		22.1×10^5^	
**Incidence rate of lung cancer** (per 10^5^ person-months) (95% C.I.)	27.1 (23.1–31.9)		21.2 (19.4–23.2)	0.009
**Death from 2004–2008 (%)**	942 (8.6)		3,291 (7.6)	<0.001
**Medications (%)**				
OCS	3,775 (34.6)		3,653 (8.4)	<0.001
LABA	5,308 (48.7)		3,455 (7.9)	<0.001
SABA	9,090 (83.4)		13,173 (30.2)	<0.001
LTRA	2,431 (22.3)		1,667 (3.8)	<0.001
Theophylline	9,999 (91.7)		29,369 (67.3)	<0.001
Statins	1,566 (14.4)		5,993 (13.7)	0.093
Aspirin	2,626 (24.1)		12,076 (27.7)	<0.001
**Sex (%)**				1.000
Men	6,329 (58.1)		25,316 (58.1)	
Women	4,575 (41.9)		18,300 (41.9)	
**Age (years, %)**				1.000
20–39	1,096 (10.1)		4,384 (10.1)	
40–59	3,367 (30.9)		13,468 (30.9)	
60–79	5,399 (49.5)		21,596 (49.5)	
≧80	1,042 (9.5)		4,168 (9.5)	
**Comorbidities (%)**				
COPD	5,665 (51.9)		5,239 (31.09)	<0.001
Diabetes	1,830 (16.8)		7,606 (17.44)	0.106
Hyperlipidemia	1,759 (16.1)		7,738 (17.74)	<0.001
Chronic kidney disease	256 (2.4)		1,033 (2.37)	0.899
Smoking-related cancers	95 (0.9)		325 (0.75)	0.178
Liver cirrhosis	128 (1.2)		559 (1.28)	0.367
Autoimmune disease	315 (2.9)		1,169 (2.68)	0.231
Atopy dermatitis	208 (1.9)		917 (2.10)	0.200
Rhinosinusitis	5,882 (53.9)		11,323 (26.0)	<0.001
No. of outpatient visits for lung disease 2 years after the index date (%)*	22.4±14.0		7.1±11.3	<0.001
≤15	3,695 (33.9)		37,600 (86.2)	<0.001
>15	7,209 (66.1)		6,016 (13.8)	
No. of inpatient visits for lung disease 2 years after the index date (%)*	0.6±1.5		0.2±0.8	<0.001
0	7,930 (72.7)		38,140 (87.4)	<0.001
≥1	2,974 (27.3)		5,476 (12.6)	
**Urbanization (%)**				<0.001
High	6,325 (58.0)		23,575 (54.1)	
Mid	3,452 (31.6)		14,285 (32.7)	
Low	1,127 (10.4)		5,756 (13.2)	

CI, confidence interval; COPD, chronic obstructive pulmonary disease; ICS, inhaled corticosteroid; LABA, long-acting inhaled beta-agonist; LTRA, leukotriene receptor antagonist; OCS, oral corticosteroid; SABA, short-acting beta-agonist; TB, pulmonary tuberculosis.

*Index date was defined as the date of initiation of ICS.

**Fig 2:**
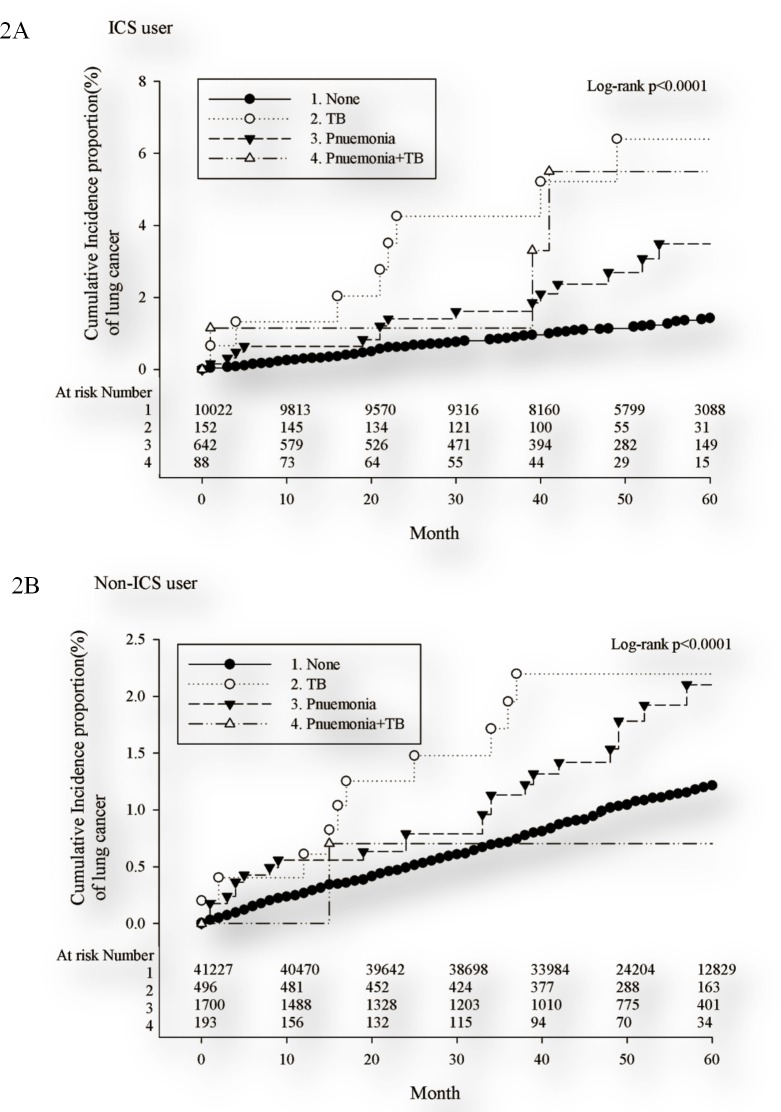
**Cumulative incidence of lung cancer for ICS users (A) and the comparison cohorts (B) stratified by pulmonary diseases in Taiwan from 2003 to 2010.** ICS, inhaled corticosteroid; TB, tuberculosis.

Kaplan-Meier plots for pneumonia, TB and pneumonia+TB stratified by ICS use are presented in Fig 2. The risks for lung cancer were greatest in patients with post-ICS pulmonary infections. The 5-year cumulative incidences for lung cancer were as follows: post-ICS TB, 6.4%, post-ICS pneumonia+TB, 5.5%, post-ICS pneumonia, 3.5%, TB, 2.2%, pneumonia, 2.1%, and control, 1.2%.

Table 2 shows the adjusted HRs of lung cancer for TB, pneumonia, and pneumonia+TB in asthmatic patients who were users and non-users of ICS compared with controls who were free of pulmonary infections. Post-ICS TB significantly increased the risk of lung cancer (hazard ratio [HR], 2.52; 95% confidence interval [CI], 1.22–5.22). The use of ICS (HR, 0.89; 95%CI, 0.69–1.14) was not associated with lung cancer in patients without lung infection. There was no significant association between lung cancer and pulmonary diseases such as TB (HR, 1.08; 95%CI, 0.57–2.03), pneumonia (HR, 0.99; 95%CI, 0.63–1.55) and pneumonia+TB (HR, 0.32; 95%CI, 0.05–2.32) among the control individuals.

**Table 2:** Hazard Ratio and 95% Confidence Intervals of Lung Cancer According to ICS and Pulmonary Disease Combinations in Patients with Asthma.

	All patients with asthma		
	HR (95% CI)		P-value
**Pulmonary disease combinations**			
None	Reference		
Only ICS	0.89 (0.69–1.14)		0.341
Only TB	1.08 (0.57–2.03)		0.815
Only Pneumonia	0.99 (0.63–1.55)		0.970
Pneumonia+TB	0.32 (0.05–2.32)		0.261
Post-ICS TB	2.52 (1.22–5.22)		0.012
Post-ICS pneumonia	1.28 (0.73–2.26)		0.389
Post-ICS pneumonia+TB	2.31 (0.84–6.38)		0.105
**Comorbidities**			
COPD	1.26 (1.05–1.53)		0.015
Smoking related cancers	1.61 (0.83–3.11)		0.158
Diabetes	0.99 (0.80–1.22)		0.912
Hyperlipidemia	0.82 (0.63–1.06)		0.127
Liver cirrhosis	0.86 (0.41–1.82)		0.695
Chronic renal disease	1.47 (0.99–2.18)		0.052
Autoimmune disease	0.87 (0.51–1.49)		0.621
Atopy dermatitis	0.60 (0.31–1.16)		0.129
Rhinosinusitis	0.91 (0.76–1.09)		0.293
**Medications**			
OCS	0.83 (0.66–1.05)		0.123
LABA	1.13 (0.90–1.42)		0.300
SABA	1.14 (0.94–1.39)		0.180
LTRA	0.99 (0.71–1.39)		0.981
Theophylline	1.31 (1.04–1.66)		0.023
Statins	1.19 (0.91–1.55)		0.209
Aspirin	1.07 (0.90–1.27)		0.451
**Sex (%)**			
Men	2.15 (1.76–2.62)		<0.001
Women	Reference		
**Age group**			
20–39	0.11 (0.03–0.46)		0.002
40–59	Reference		
60–79	3.84 (2.90–5.07)		<0.001
≧80	6.12 (4.43–8.45)		<0.001
No. of outpatient visits for lung disease 2 years after the index date*			
≤15	Reference		
>15	1.28 (1.04–1.58)		0.019
No. of inpatient visits for lung disease 2 years after the index date*			
0	Reference		
≥1	0.95 (0.74–1.21)		0.654
**Urbanization**			
High	Reference		
Mid	1.10 (0.92–1.31)		0.287
Low	1.06 (0.83–1.34)		0.658

CI, confidence interval; COPD, chronic obstructive pulmonary disease; HR, hazard ratio; ICS, inhaled corticosteroid; LABA, long-acting inhaled beta-agonist; LTRA, leukotriene receptor antagonist; OCS, oral corticosteroid; SABA, short-acting beta-agonist; TB, pulmonary tuberculosis.

*Index date was defined as the date of initiation of ICS.

## Discussion

ICSs are the mainstay treatment to control airway inflammation and acute exacerbations in patients with asthma [21]. They also suppress the immune, thereby increasing the risk of TB and pneumonia [15, 16]. TB and pneumonia cause lung inflammation and have also been associated with lung cancer [3, 5, 7]. However, the impact of post-ICS TB and pneumonia on lung cancer has not been addressed. In this study, a higher risk of lung cancer was observed in patients with post-ICS TB.

In a case-control study to investigate lung cancer risk in non-smoking women (412 cases and 1,253 controls) in the United States, the increased risks associated with previous lung disease were observed in patients with asthma (odds ratio [OR], 1.67; 95%CI, 1.1–2.5) [22]. A case-control study of female lifetime nonsmokers showed an increased risk of lung cancer in asthmatic patients (OR, 4.78; 95%CI, 1.23–18.63) [23]. An increased risk of lung cancer was reported in a Swedish cohort of 92,986 patients with a hospital-discharge diagnosis of asthma [24]. The standardized incidence ratio was 1.51 (95% CI, 1.38–1.65) in men and 1.78 (95% CI, 1.55–2.03) in women compared with that of the national population. Increased risks of lung cancer have also been reported in Taiwanese men and women [6]. The HRs were 1.36 (95%CI, 1.30–1.41) and 1.26 (95%CI, 1.18–1.34), respectively. In a meta-analysis, asthma has been associated with an increase in the overall relative risk (RR) of lung cancer (RR, 1.28; 95%CI, 1.16–1.41) [25].

Lee et al. analyzed first-time adult users of ICS (9,177 cases and 37,048 controls) and noted that ICS use had a significant linear association with a decreased lung cancer incidence (OR, 0.79; 95%CI, 0.69–0.90) [12]. In a previous study, analyses were conducted on patients newly diagnosed with adult-onset asthma (i.e., 2,117 regular ICS users and 17,732 non-ICS users) [26]. The findings showed no protective effect of regular ICS use on lung cancer. In our study, there was no increased risk of lung cancer in ICS users who were free of pulmonary infections.

There are multiple anti-inflammatory and immunosuppressive effects of glucocorticoids. They suppress macrophage differentiation, production of cytokines, and tumoricidal and microbicidal activities of activated macrophages and inhibit T-cell activation [27]. The joint statement of the American Thoracic Society and the Centers for Disease Control and Prevention acknowledges that taking the equivalent of ≧15 mg/day of prednisone administered for 1 month or more is a risk factor for TB [28]. In a retrospective cohort study of 616 patients with COPD, Kim and colleagues showed that ICS use was an independent risk factor for the development of pulmonary TB in patients who had a normal chest radiograph (HR, 9.08; 95% CI, 1.01–81.43) and those who had radiologic sequelae of prior pulmonary TB (HR, 24.95; 95% CI, 3.09–201.37) [29]. In a nested case-control study based on the Korean national claims database with 4,139 TB cases and 20,583 controls, ICS use had a significant linear association with increased risk of TB (OR, 1.20; 95% CI, 1.08–1.34) [15]. Chung et al. analyzed samples from the NHIRD and found a multiplicative-increase in the risk of TB in ICS and OCS users compared with their non-user counterparts (OR, 4.31; 95% CI, 3.39–5.49) [30]. However, few studies have addressed the association between ICS use and pneumonia in patients with asthma. In a nested case-control study, asthmatic patients who received ICSs were at increased risks for pneumonia and lower respiratory tract infections [31]. The greatest risk was in patients who received higher doses of ICS. Conversely, in a meta-analysis of clinical randomized trials of asthmatics using ICSs (budesonide and fluticasone propionate), patients did not have an increased risk of pneumonia even at higher doses or among the different ICSs [32]. In our study, there were higher rates of TB, pneumonia and pneumonia+TB in ICS users than their non-user counterparts.

In a study involving 4,480 patients with newly diagnosed TB, Yu et al. showed that there was an increased risk of lung cancer in TB patients [33]. The adjusted HR was 3.32 (95% CI, 2.70–4.09). Pneumonia has also been associated with an increased risk of lung cancer (HR, 4.24; 95%CI, 3.96–4.55) [7]. A meta-analysis showed that previous history of pneumonia and TB conferred RRs of 1.43 (95% CI, 1.22–1.68) and 1.76 (95% CI, 1.49–2.08) for lung cancer, respectively [34]. When restricted to nonsmokers, it remained significant for pneumonia (RR, 1.36; 95% CI, 1.10–1.69) and TB (RR, 1.90; 95% CI: 1.45–2.50).

In this study, there was a close association between post-ICS TB and lung cancer in patients with asthma. Jian and colleagues reported a stronger association between coexisting asthma/TB and lung cancer in men and women [5]. Their HRs were 2.12 (95%CI, 1.59–2.83) and 2.21 (95%CI, 1.28–3.81), respectively. Biologically, the effects of post-ICS TB on lung cancer may be explained by asthma and TB-related chronic inflammatory processes of lung, corticosteroids-induced compromised immune clearance of *Mycobaterium tuberculosis* and malignant cells that predispose to lung cancer. However, this study found no statistically significant additive effect of post-ICS pneumonia and TB on lung cancer (HR, 2.31; 95% CI, 0.84–6.38; p = 0.105). The prevalence of pneumonia+TB in ICS and non-ICS users was 0.8 (88/10,904) and 0.5% (193/43,616), respectively. Lung cancer risk was found to be higher in patients with more than one of the lung diseases (TB, pneumonia, chronic bronchitis and asthma) [23]. This may have been due to the relatively small sample size. Nonetheless, such findings warrant further investigations.

In the US, the National Lung Screening Trial (NLST) included asymptomatic individuals aged 55 to 74 years who had a history of at least 30 pack-years of smoking and who were either current smokers or had been smokers within the previous 15 years [35]. Results showed a 20% relative reduction in lung cancer-specific mortality with the use of 3 screenings (at 1-year intervals) with low-dose computed tomography (CT) than with the use of chest radiography. Wu et al. retrospectively analyzed the medical records of 1,763 asymptomatic individuals who underwent self-paid low-dose CT in Taiwan [36]. Results showed that 8.4% (148/1,763) of study individuals were eligible for lung cancer screening based on the NLST criteria and only one participant was diagnosed with lung cancer. Twenty-four individuals (24/1615) who did not meet NLST eligible criteria were diagnosed with lung cancer. They also found that female and a family history of lung cancer were the 2 most important predictors of lung cancer. Chen et al. retrospectively reviewed the medical records of 3,339 asymptomatic individuals who received annual medical examinations and reported 34 cases of lung cancer [37]. The overall cancer detection rate was 1.02%. Individuals who were younger than 50 years with a positive family history of all types of cancers in first-degree relatives were at elevated risk of the disease (detection rate, 6.2%; 8/129). From above studies, a family history of cancer was the most important predictor of lung cancer in Taiwan.

Studies to investigate lung cancer incidence in Taiwanese smokers and non-smokers are scarce. The population in Taiwan is approximately 23 million and the smoking rate among adults in Taiwan has decreased from 23.0% (men, 39.2; women, 5.4%) in 2007 to 19.5% (33.6 and 4.6%) in 2011, respectively [38]. Smoking history has been officially included in the TCRD since 2011. Among patients diagnosed with lung cancer in 2011 and 2012 (n = 21,536), 42.4% were smokers [39]. The age-standardized incidence rate of lung cancer in Taiwan in 2011 was 34.0/100,000 (men, 45.0/100,000; women, 25.2/100,000) [40]. In this study, the 5-year cumulative incidence of lung cancer in patients with post-ICS TB was 6.4%. Post-ICS TB was an important predictor for lung cancer.

Established risk factors for lung cancer include tobacco smoke, genetic factor, alcohol, and occupation exposure. Smoking is the major risk factor for lung cancer. However, most of the female lung cancer patients in Taiwan were non-smokers [41]. Evidence shows that lung cancer tends to aggregate in families [42, 43]. A positive association has been reported between all cancer history of first-degree relatives and the risk of lung cancer among lifetime nonsmokers in Hong Kong (OR, 2.14; 95%CI, 1.30–3.54) [23]. A family history of lung cancer has been reported as a significant predictor of lung cancer for non-smoking females in Taiwan (OR, 5.7; 95% CI, 1.9–16.9) [44]. This shows that genetic susceptibility might influence the risk of lung cancer. Alcohol consumption has been associated with a modest increase in lung cancer risk (i.e., for ≥7 drinks/day, the HR = 1.11; 95% CI, 1.00–1.24) compared with nondrinking [45]. Among the possible causes of lung cancer, occupational risk factors are potentially preventable. Lung cancer risks were higher in workers who were employed in asbestos-processing industries, metal foundries and petrochemical industries [2, 46].

The strengths of this study were numerous. First, our study was a retrospective cohort study which included large sample size and long follow-up duration. In addition, the temporal relationship between post-ICS pulmonary infections and lung cancer is difficult to evaluate in case–control studies. Second, there was the completeness of cancer case ascertainment, hence allowing the little possibility for recall and selection bias. Nevertheless, this study had some limitations. First, detection bias might have been possible because of the frequent hospital visits which might have led to a higher detection rate of TB and early-stage lung cancer. Second, pulmonary comorbidities may significantly mask symptoms and delay the diagnosis of lung cancer or may even prevent a full diagnostic evaluation with the proper staging of the disease. We excluded patients who were diagnosed with TB or pneumonia before or within 3 months after the index date. Third, the national databases do not contain detailed information regarding smoking, alcohol, radon exposure, occupational exposures, diet preference, and family history, all of which may be risk factors for lung cancer.

## Conclusions

We found that post-ICS TB conferred a higher risk of lung cancer than exposure to pulmonary diseases among non-users of ICS. Because of the high mortality associated with lung cancer, screening programs should include post-ICS TB.

## Abbreviations

CI: confidence interval
COPD: chronic obstructive pulmonary disease
CT: computed tomography
HR: hazard ratio
ICD-9-CM: International Classification of Diseases, Ninth Revision, Clinical Modification code
ICS: inhaled corticosteroid
LABA: long-acting inhaled β2 agonists
LTRA: leukotriene receptor antagonist
NDRD: National Death Registry Database
NHIRD: National Health Insurance Research Database
NLST: National Lung Screening Trial
OCS: oral corticosteroid
OR: odds ratio
RR: relative risk
SABA: short-acting inhaled β2 agonists
TB: tuberculosis
TCRD: Taiwan Cancer Registry Database
